# Comparative global B cell receptor repertoire difference induced by SARS-CoV-2 infection or vaccination via single-cell V(D)J sequencing

**DOI:** 10.1080/22221751.2022.2105261

**Published:** 2022-08-11

**Authors:** Bing He, Shuning Liu, Mengxin Xu, Yunqi Hu, Kexin Lv, Yuanyuan Wang, Yong Ma, Yanmei Zhai, Xinyu Yue, Lin Liu, Hongjie Lu, Siwei Zhou, Pengbin Li, Guoqin Mai, Xiaoping Huang, Chenhang Li, Shifeng Chen, Shupei Ye, Pingsen Zhao, Yuedong Yang, Xinhua Li, Yusheng Jie, Mang Shi, Jingyi Yang, Yuelong Shu, Yao-Qing Chen

**Affiliations:** a School of Public Health (Shenzhen), Shenzhen Campus of Sun Yat-sen University, Shenzhen, People’s Republic of China; b School of Public Health (Shenzhen), Sun Yat-sen University, Guangzhou, People’s Republic of China; c Department of Respiratory and Critical Care Medicine, The 74(th) Group Army Hospital, Guangzhou, People’s Republic of China; d SSL Central Hospital of Dongguan City, Dongguan, People’s Republic of China; e Laboratory for Diagnosis of Clinical Microbiology and Infection, Medical Research Center, Yuebei People’s Hospital, Shantou University Medical College, Shaoguan, People’s Republic of China; f School of Data and Computer Science, Sun Yat-sen University, Guangzhou, People’s Republic of China; g Department of Infectious Diseases and Key Laboratory of Liver Disease of Guangdong Province, The Third Affiliated Hospital of Sun Yat-sen University, Guangzhou, People’s Republic of China; h Department of Infectious Diseases, The Third Affiliated Hospital of Sun Yat-sen University, Guangzhou, People’s Republic of China; i The Centre for Infection and Immunity Studies, School of Medicine, Shenzhen Campus of Sun Yat-sen University, Sun Yat-sen University, Shenzhen, People’s Republic of China; j Vaccine and Immunology Research Center, Translational Medical Research Institute, Shanghai Public Health Clinical Center, Fudan University, Shanghai, People’s Republic of China; k Ministry of Education, Key Laboratory of Tropical Disease Control (Sun Yat-sen University), Guangzhou, People’s Republic of China

**Keywords:** BCR repertoire, single-cell RNA sequencing, COVID-19, SARS-CoV-2 vaccination, SARS-CoV-2 infection, inactivated vaccine

## Abstract

Dynamic changes of the paired heavy and light chain B cell receptor (BCR) repertoire provide an essential insight into understanding the humoral immune response post-SARS-CoV-2 infection and vaccination. However, differences between the endogenous paired BCR repertoire kinetics in SARS-CoV-2 infection and previously recovered/naïve subjects treated with the inactivated vaccine remain largely unknown. We performed single-cell V(D)J sequencing of B cells from six healthy donors with three shots of inactivated SARS-CoV-2 vaccine (BBIBP-CorV), five people who received the BBIBP-CorV vaccine after having recovered from COVID-19, five unvaccinated COVID-19 recovered patients and then integrated with public data of B cells from four SARS-CoV-2-infected subjects. We discovered that BCR variable (V) genes were more prominently used in the SARS-CoV-2 exposed groups (both in the group with active infection and in the group that had recovered) than in the vaccinated groups. The VH gene that expanded the most after SARS-CoV-2 infection was IGHV3-33, while IGHV3-23 in the vaccinated groups. SARS-CoV-2-infected group enhanced more BCR clonal expansion and somatic hypermutation than the vaccinated healthy group. A small proportion of public clonotypes were shared between the SARS-CoV-2 infected, vaccinated healthy, and recovered groups. Moreover, several public antibodies had been identified against SARS-CoV-2 spike protein. We comprehensively characterize the paired heavy and light chain BCR repertoire from SARS-CoV-2 infection to vaccination, providing further guidance for the development of the next-generation precision vaccine.

## Introduction

The distinctive severe acute respiratory syndrome coronavirus 2 (SARS-CoV-2), which caused the 2019 coronavirus disease (COVID-19), has posed a severe threat to global health. Vaccines are the most effective measures to prevent and control the SARS-CoV-2 pandemic [1]. Eleven SARS-CoV-2 vaccines were granted emergency use listing by WHO (https://covid19.trackvaccines.org/agency/who/), including BNT16b2, mRNA-1273, ChAdOx1 nCoV-19 and BBIBP-CorV vaccines and others. B cells are critical for producing antibodies and conferring protective immunity to SARS-CoV-2 infection and vaccination [2]. Naïve B cells recognize various antigens of a pathogen at the early phase of infection and then undergo affinity maturation in a germinal center through somatic hypermutation (SHM) and class-switch recombination (CSR). Mature B cells can react strongly to foreign antigens, resulting in B cell stimulation, clonal expansion, and ultimately, the secretion of high-affinity antibodies in the blood [3]. During B cell development, single variable (V), diversity (D), and joining (J) genes are selected from multiple distinct copies and imprecisely joined to create a B cell receptor (BCR) [4].

Global features of the BCR repertoire have been studied by high-throughput bulk RNA sequencing of immunoglobulin heavy chain (IgH) genes [5–7]. IgH BCR repertoires studies showed different usage of V gene and immunoglobulin isotype switch post several types of COVID-19 vaccination or virus infection in subjects [8]. The usage of IGHV1-24 was increased in the severely infected group during the early stage, while no similar trend was observed post-BNT162B2 SARS-CoV-2 vaccination [8]. V genes usage in the IGHV1-69D, IGKV1D-39, and IGLV5-45 were observed to increase after the Ad5-based recombinant SARS-CoV-2 vaccine (Ad5-nCoV, trade name: Convidecia™) [9]. For the inactivated COVID-19 vaccination, IGHV1-18, IGHV3-11, IGHV3-23, IGHV4-34 and IGHV4-59 were predominantly used in the total IgH repertoire [10]. IgM and IgA accounted for the highest ratio post inactivated COVID-19 vaccination, while IgG post mRNA vaccination [8,10]. However, knowledge about the endogenous pairing of heavy and light chain repertoires is lost in most studies.

As SARS-CoV-2 natural infection induces stronger and more long-lasting protective immune responses than vaccination, the BCR repertoire can be expected to be different between them, which can affect the efficacy of both the acute response to viral infection and the quality and longevity of the memory response [8]. However, we still lack an understanding of the overall landscape of BCR repertoires in subjects with on-going SARS-CoV-2 infection and in previously recovered/naïve subjects who received an inactivated SARS-CoV-2 vaccine, especially at the single-cell level. To learn those insights, we tracked the development of paired heavy and light chain BCR repertoires in longitudinal samples collected from six healthy recipients of inactivated SARS-CoV-2 vaccine (BBIBP-CorV), five people who received the BBIBP-CorV vaccine after having recovered from COVID-19, five unvaccinated COVID-19 recovered people and then integrated with public data of B cells from four SARS-CoV-2 infected patients. Via single-cell V(D)J sequencing, we mapped 163,161 heavy and light paired B cells from the above groups. Preferential V genes usage, immunoglobulin isotypes, clonal expansion, SHM, and public antibody clonotypes were characterized in this study. Our study provided detailed insights on BCR repertoire signatures in the varying nature landscape of SARS-CoV-2 exposure, contributing to a better understanding of the humoral immune response and optimizing the next generation of infectious disease vaccines.

## Material and methods

### Study design and participants

Six healthy donors with three shots of inactivated SARS-CoV-2 vaccine (BBIBP-CorV), five people who received the BBIBP-CorV vaccine after having recovered from COVID-19 and five unvaccinated COVID-19 recovered patients were enrolled in this study. Four SARS-CoV-2 infected patients’ information from public data was integrated with the above data [11]. All study procedures were approved by the Research Ethics Committee of School of Public Health (Shenzhen), Sun Yat-sen University, China.

### Cell hashing

To avoid possible sample-specific batch effects, cell hashing was used to distinct samples. It uses a series of oligo-labelled antibodies against CD298 and β2 microglobulin with different barcodes to uniquely label the cells from different individuals, and then they were pooled in one scRNA-seq run.

Sorted cells from those above subjects were pooled into one tube and stained with a mixture of oligo-labelled antibodies against target surface proteins. The detailed methods were generated as previously described [12].

### Single-cell V(D)J sequencing

Peripheral blood mononuclear cells (PBMCs) were isolated from the blood of the above samples using a human lymphocyte separation medium. Cells were stained and mixed with DNA-barcoded antigens and other fluorescent antibodies. After sorted by FACS with a MoFlo Astrios EQ Flow Cytometer (Beckman). All B cells were loaded on a 10X Chromium A Chip. Single-cell lysis and RNA first-strand synthesis were performed using 10X Chromium Single-cell 50 Library & Gel Bead Kit according to the manufacturer’s protocol. The following RNA and V(D)J library preparation were performed according to the manufacturer’s protocol (Chromium Single-cell V(D)J Reagent Kits, 10X Genomics). The detailed methods were generated as previously described[12].

### V(D)J sequencing analysis

Single-cell V(D)J sequencing data were initially processed with Cell Ranger (v.5.0.0) vdj pipeline against the GRCh38 reference genome. BCR contigs contained in “filtered_contigs.fasta” and “filtered_contig_annotations.csv” from Cell Ranger vdj output were consequently preprocessed by the dandelion Python package [13]. Dandelion was performed to reannotate v(d)j sequence with “igblastn” using the IMGT database as a reference and quantify SHM. BCR Contigs with only one heavy chain and one light chain were continued for clonotypes analysis using scirpy package(v.0.10.1) [14]. Clonotypes were defined according to whether the v(d)j nucleotide sequences were identified. We used two different definitions for analysis of shared clonotypes: similar CDR3 amino acid sequence with and without the same VJ gene usage in both heavy and light chains [15]. The BLOSUM62 matrix was applied to all of the repertoires to calculate the distance between sequences based on pairwise sequence alignment by scirpy.pp.ir_dist with cut-off = 10. Then scirpy was performed to define clonotype clusters or shared clonotypes that have different CDR3 nucleotide sequences but might recognize the same antigen because they have the same or similar CDR3 amino acid sequence. Shared clonotypes (shared CDR3 only) were shown with clonotype network plots. Upset plots were drawn to exhibit shared clonotypes with the same VJ usage. Besides, clonal expansion, VJ genes usage, isotype, etc. were also taken for analysis.

### Monoclonal antibody expression and purification

Antibodies were generated as previously described [16,17]. The convergent paired monoclonal antibodies (mAbs) were synthesized and cloned into expression vectors containing the IgG constant regions of the human using SalI and XhoI restriction enzymes (Monad MF02201 and MF03001). IgG mAbs were expressed by transfecting HEK293T cells with equal amounts of heavy- and light-chain plasmids using polyethylenimine (PEI) and cultured for 5 days. The supernatant was collected and purified using protein A agarose beads.

### Enzyme-linked immunosorbent assay (ELISA)

High-protein binding microtiter plates (Costar) were coated with 2 µg/ml SARS-CoV-2 S1, and S2 recombinant protein in PBS overnight at 4°C, respectively. After blocking with 3% BSA in 1 × PBS, serially diluted (1:3 ratio) mAbs starting at 10 µg/ml were added to the plates and incubated for 1 h at 37°C. Plates were washed six times with PBST and then incubated with HRP (horse radish peroxidase) conjugated goat anti-human IgG (or IgA and IgM) (JACKON). The plate was developed with Super Aquablue ELISA substrate (eBiosciences). Absorbance was measured at 405 nm on a microplate spectrophotometer (BioTek).

### Statistical analysis

Statistical analyses were performed with R soft (R version 4.1.1) and Python (version 3.5). Fisher’s exact test was used to assess differences in the use of VH gene segments and immunoglobulin isotypes. Two-side t-tests or Wilcoxon rank-sum tests were used to analyze differences in CDRH3 length and SHM rate among different groups. Spearman’s rank correlation was used to analyze the correlation between CDRH3 length and immunoglobulin isotype proportion. In all analyses statistical significance was tested, significance was defined as *p value < 0.05; **p value < 0.01; ***p value < 0.001 and **** p value < 0.0001; not significant (ns): *p*-value >0.05.

## Results

### Preferential BCR V gene segments usage bias was more prominent in the SARS-CoV-2 infected group than that in the vaccinated healthy group

To compare the global difference of paired BCR repertoire between SARS-CoV-2 infection and vaccination, we collected PBMCs from six healthy recipients with three shots of BBIBP-CorV (the Vaccinated Healthy group), and those people in 0 d before vaccination (the Healthy group) were considered as the baseline, five people who had recovered from COVID-19 and had received a single dose of SARS-CoV-2 inactivated virus vaccine (the Vaccinated Recovered group), and five COVID-19 recovered people (the Unvaccinated Recovered group), respectively.

**Figure 1.**
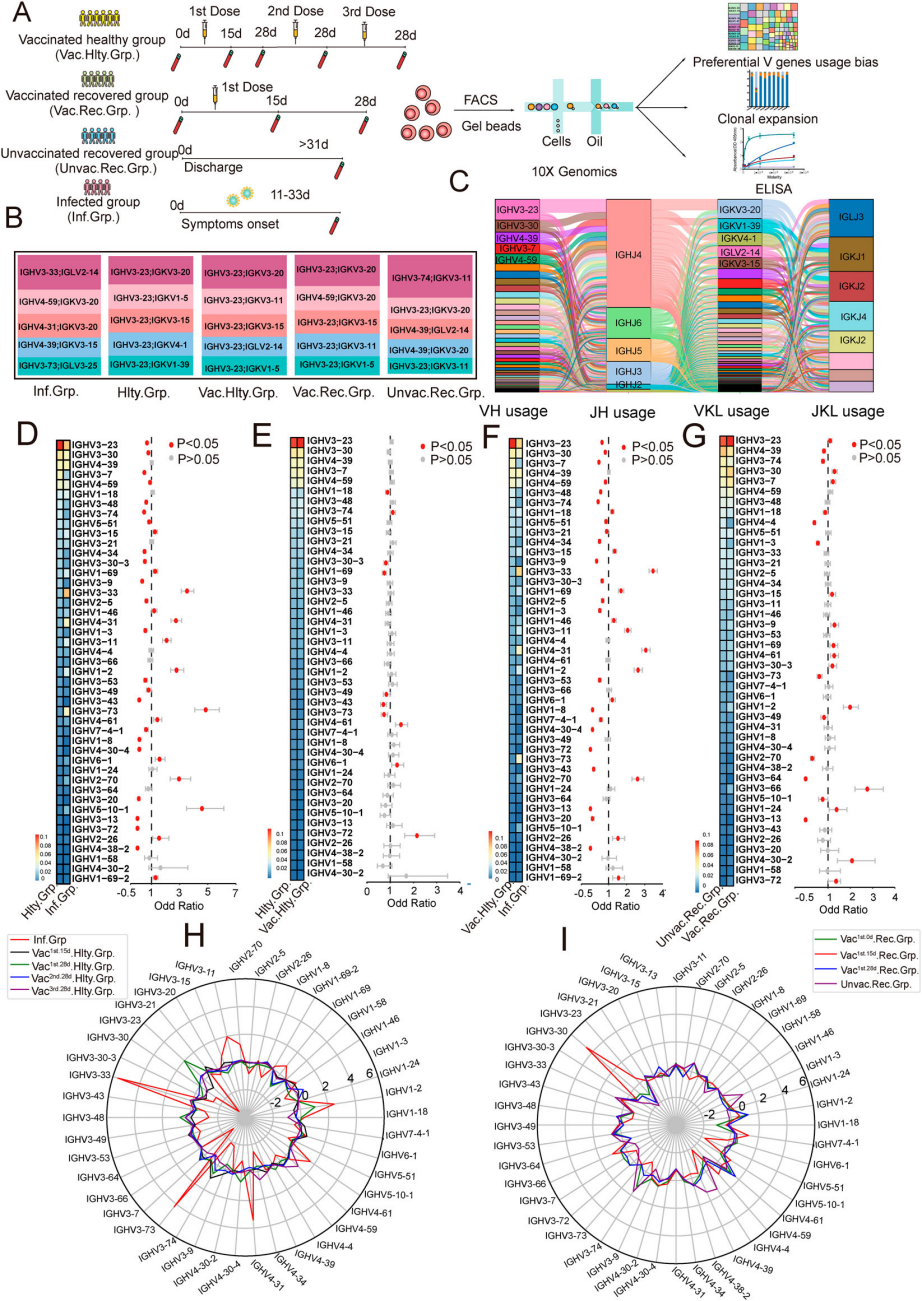
Preferential V Gene Segments Usage of BCR in SARS-CoV-2 Infection and Vaccination. (A) Schematic overview of the 10X Genomics single-cell V(D)J sequencing process. The global BCR repertoire characterization in different nature of SARS-CoV-2 exposure. (B)The distribution of the top 5 paired heavy and light chains in SARS-CoV-2-infected (Inf.Grp.), healthy (Hlty.Grp.), vaccinated healthy (Vac.Hlty.Grp.), vaccinated recovered (Vac.Rec.Grp.) and unvaccinated recovered groups (Unvac. Rec.Grp.). (C) Sankey diagram shows total BCR-specific heavy and light VJ pairs that occurred more than once. (D–G) The frequency and differential analysis of VH gene segments between healthy and infected groups (D), healthy and vaccinated healthy groups (E), vaccinated healthy and infected groups (F), unvaccinated recovered and vaccinated recovered groups (G). The colours represent the *p*-value of the significant positive or negative. Red: *p* < 0.05, grey: *p* > 0.05. Fisher’s exact test, *p*-value less than 0.05, was considered to be statistically significant. (H) The polar plot shows the relative changes of VH genes usage in SARS-CoV-2-infected (Inf.Grp), vaccinated^1st.15d^ healthy (Vac^1st.15d^.Hlty.Grp.) vaccinated^1st.28d^ healthy (Vac^1st.28d^.Hlty.Grp.), vaccinated^2nd.28d^ healthy (Vac^2nd.28d^.Hlty.Grp.)and vaccinated^3rd.28d^ healthy(Vac^3rd.28d^.Hlty.Grp.) groups. The VH gene usage proportion of the healthy group (Hlty.Grp.) was considered as the baseline. The relative changes in VH gene usage were calculated by the following method: the proportion of VH gene at a certain time point minus above the baseline. (I) The polar plot shows the relative changes of VH genes usage in vaccinated^1st.0d^ recovered (Vac^1st.0d^.Rec.Grp.), vaccinated^1st.15d^ recovered (Vac^1st.15d^.Rec.Grp.), vaccinated^1st.28d^ recovered (Vac^1st.28d^.Rec.Grp.) and unvaccinated recovered (Unvac.Rec.Grp.) groups. The calculation method of relative changes in VH genes usage was the same as in Figure 1(H).

We sorted the sub-populations of B cells (CD19^+^ CD27^+^ memory B cells, and CD19^+^ CD27^high^ CD38^high^ plasma cells) (Figure S1). The sorted cells were then subjected to 10X Chromium, and then single-cell V(D)J sequencing was performed (Figure 1(A)). Moreover, the BCR repertoire from four SARS-CoV-2-infected patients (the Infected group), collected from public data, were integrated into this study. The detailed basic information of the above groups is shown in Tables S1–S4. The sorting strategy of the above four infected samples was similar to our study [11].

To study the preferential BCR V genes usage bias by SARS-CoV-2 infection or a SARS-CoV-2 inactivated vaccine at a single-cell level, 163,161 paired heavy and light chains BCR clones were obtained, including 22678, 81236, 33770, 13121 and 12356 heavy and light pair BCR clones in the infected, vaccinated healthy, vaccinated recovered, unvaccinated recovered and healthy groups, respectively. The preferential usage bias of the top 5 paired BCR clones are shown in Figure 1(B). The top five paired BCR clonotypes in the infected group were different from that in the healthy group. The top five paired clonotypes in the vaccinated healthy group were still similar to that of the healthy group (Figure 1(B)). Among these, the first and third highest frequency paired clonotypes were the same (IGHV3-23/IGKV3-20, IGHV3-23/IGKV3-15). While the second highest frequency paired clonotypes in the infected group and the vaccinated recovered group were the same (IGHV4-59/IGKV3-20). The most frequent heavy and light VJ pair in all groups was IGHV3-23-IGHJ4-IGKV3-20-IGKJ1, followed by IGHV3-23-IGHJ4-IGKV1-5-IGKJ1 and IGHV3-23-IGHJ4-IGKV3-11-IGKJ4 (Figure 1(C)).

In most antibodies, the heavy chain is generally considered to play a major role in antigen-binding interactions in most antibodies. Hence, we explored the usage bias of VH gene segments. Compared with the healthy group, fourteen VH gene segments (fourteen heavy chains) significantly increased in the infected group (*p*< 0.05) (Figure 1(D)). IGHV3-33 was the dominant VH gene expanded post-infection, followed by IGHV3-73 and IGHV4-31(Figure 1(D)). However, in the vaccinated healthy group, only four VH gene segments were increased (*p* < 0.05), in which IGHV3-74 was the dominant expanded VH gene (Figure 1(E)). Compared with the vaccinated healthy group, the infected group had remarkably increased usage of twelve VH gene segments (*p *< 0.05) (Figure 1(F)). Thirteen VH gene segment usage increased in the vaccinated recovered group compared to the unvaccinated recovered group (*p* < 0.05) (Figure 1(G)).

Then we explored the longitudinal VH genes segment usage bias dynamics of SARS-CoV-2 infection and vaccination, by analyzing relative changes in VH gene usage. When considering the VH gene usage proportion of 0 day before vaccination as the baseline, relative changes in VH gene usage could be calculated. As Figure 1(H) shows, VH gene segment usage is extremely different between infection and vaccination, the proportions of some VH genes, such as IGHV3-33, IGHV4-31, IGHV3-73, were sharply increased, while IGHV3-23 and IGHV3-7 were relatively decreased. IGHV3-30 accounted for the highest proportion in 15 days post recovered people first shot with the inactivated vaccine (vaccinated^1st.15d^ recovered group), then go back to steady state at 28 days post-vaccination (the vaccinated^1st.28d^ recovered group, Figure 1(I)). Biased relative changes in VK/VL-gene usages were conspicuous to observe between the infected and vaccinated healthy groups (Figure S2A–C), the unvaccinated recovered and vaccinated recovered groups (Figure S2B–D).

Taken together, these results highlighted V genes usage bias in the SARS-CoV-2-infected group was more prominent than that in the vaccinated healthy group. The different usage bias of VH/VL gene segments indicated antibody specificity elicited by infection and vaccination were very different. These findings indicated that it can distinguish the sample’s immune status by the V gene usage.

### Immunoglobulin isotypes were dominated by IgA and IgG post-infection, and by IgM post-inactivated vaccine

**Figure 2.**
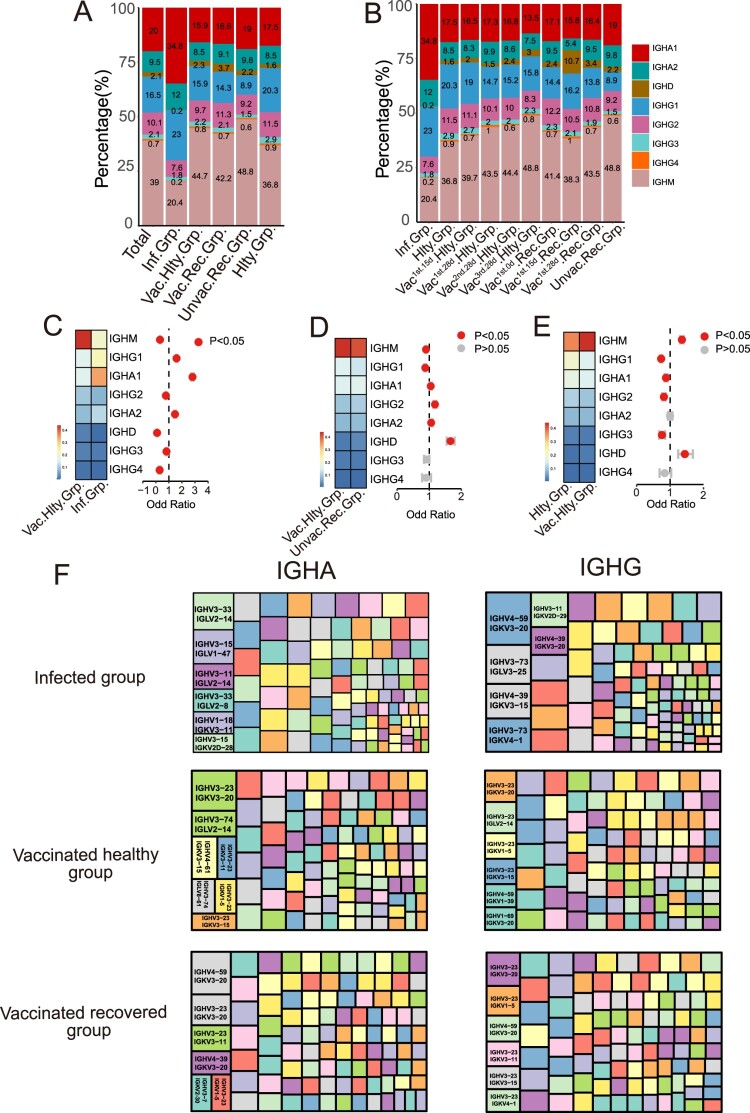
Immunoglobulin Isotypes Signature Analysis of SARS-CoV-2 Infection and Vaccination. (A) The bar graph shows immunoglobulin isotype distribution in Total, SARS-CoV-2 infected, vaccinated healthy, vaccinated recovered, unvaccinated recovered and healthy groups. (B) The bar graph shows immunoglobulin isotype distribution in SARS-CoV-2 infected, healthy, vaccinated^1st.15d^ healthy, vaccinated^1st.28d^ healthy, vaccinated^2nd.28d^ healthy, vaccinated^3rd.28d^ healthy, vaccinated^1st.0d^ recovered, vaccinated^1st.15d^ recovered, vaccinated^1st.28d^ recovered and unvaccinated recovered groups. (C–E) The frequency and differential analysis of immunoglobulin isotypes between vaccinated healthy and infected groups(C), vaccinated healthy and unvaccinated recovered groups(D) and healthy and vaccinated healthy groups(E). The colours represent the *p*-value of the significant positive or negative, red: *p* < 0.05, grey: *p* > 0.05. Fisher’s exact test, *p*-value less than 0.05 was considered to be statistically significant. (F) Combination of V genes of heavy chain and light chain. The combination of V genes of heavy chain and light chain of IgA and IgG in infected vaccinated healthy and vaccinated recovered groups.

After activation, B cells may undergo CSR to change the isotype of their BCR from IgM and IgD to IgG, IgA, or IgE [3,18]. We first compared the usage frequency of different immunoglobulin isotypes among SARS-CoV-2 infection and vaccination. IgM presented the highest proportion in total BCR repertoire (39%) in all groups, except the infected group (Figure 2(A,B)). The proportions of IgA1 and IgG1 were increased in the infected group compared to the vaccinated healthy group (*p* < 0.05) (Figure 2(C)). The usage of IgA1, IgA2 and IgG2 increased significantly in the vaccinated recovered group compared to the vaccinated healthy group (*p* < 0.05) (Figure 2(D)). However, there were no statistical differences in IgA2 and IgG4 between the vaccinated healthy and healthy groups (Figure 2(E)). Inactivated vaccine induced an infirm immunoglobulin isotype switch response, which is similar to that of the healthy group.

Next, we analyzed the paired heavy and light chain usage bias of different immunoglobulin isotypes in the infected, vaccinated healthy and vaccinated recovered groups. For IgA isotype, the paired heavy and light chain IGHV3-33/IGLV2-14, IGHV3-23/IGKV3-20 and IGHV4-59/IGKV3-20 were the dominant usage BCR gene in the infected, vaccinated healthy and vaccinated recovered groups, respectively (Figure 2(F)). For IgG isotype, the dominant usage was IGHV4-59/IGKV3-20, IGHV3-23/IGKV3-20 and IGHV3-23/IGKV3-20, respectively (Figure 2(F)). Altogether, immunoglobulin isotypes class switching following SARS-CoV-2 vaccination was less pronounced than after infection. The clonal expansion was most prominent in IgA post-infection but IgM after inactivated vaccine. The increasing percentage of IgA indicates that IgA may be synthesized and migrate widely to the respiratory tract and or other mucosal sites to play an early immune function to clear virus [19]. This informed the next-generation vaccine strategy should target a strong mucosal IgA response.

### Greater breadth of BCR clonal expansion following SARS-CoV-2 infection compared with the vaccination

**Figure 3.**
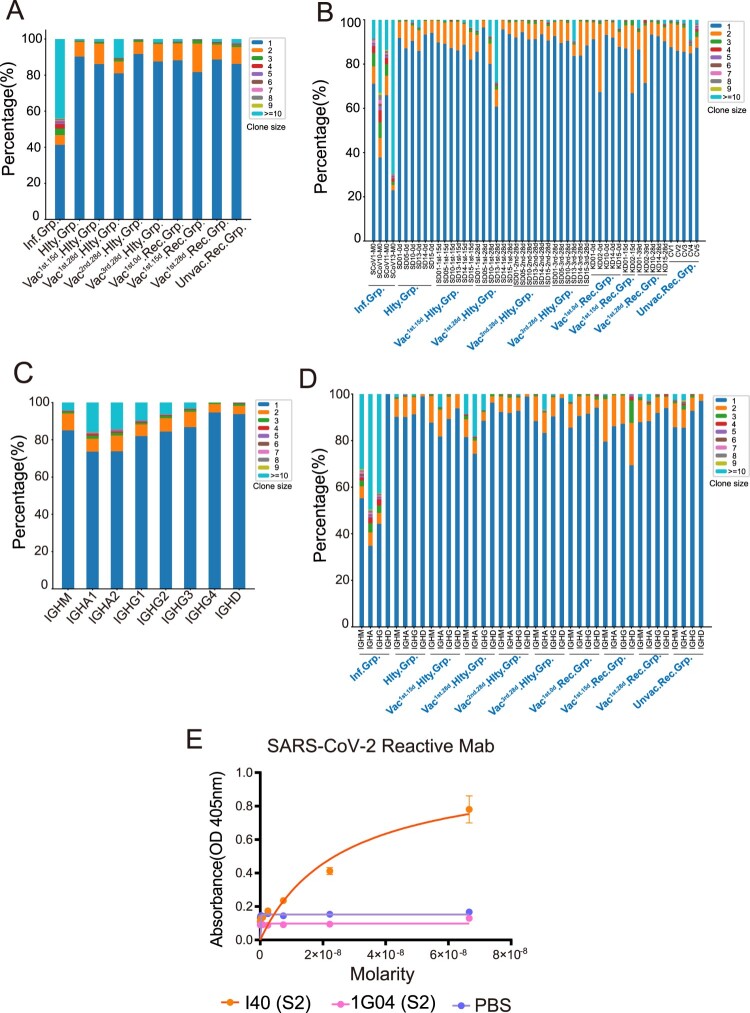
BCR Clonal Expansion Following SARS-CoV-2 Infection and Vaccination. (A) The Bar plot shows clonal expansion distribution in SARS-CoV-2 infected, healthy, vaccinated^1st.15d^ healthy, vaccinated^1st.28d^ healthy, vaccinated^2nd.28d^ healthy, vaccinated^3rd.28d^ healthy, vaccinated^1st.0d^ recovered, vaccinated^1st.15d^ recovered, vaccinated^1st.28d^ recovered and unvaccinated recovered groups. The different colours represent the clone size. (B) The Bar plot shows the clonal expansion distribution in each sample. (C) The Bar plot shows clonal expansion distribution in different immunoglobulin isotypes, including IgM, IgA1, IgA2, IgG1, IgG2, IgG3, IgG4 and IgD. (D) The Bar plot shows the clonal expansion distribution of different immunoglobulin isotypes in different groups. (E) Binding of top1 dominant antibody to SARS-CoV-2 S2 antigen by ELISA.

Clonal expansion is a sign of positive selection. To compare the differences in BCR clonal expansion after SARS-CoV-2 infection and vaccination, we analyzed the clone size distribution in all samples. The SARS-CoV-2-infected group had higher proportion of expanded clones (>50%; clone size ≥ 2) than the vaccinated by SARS-CoV-2-inactivated virus vaccine (<20%, Figure 3(A)). All the four SARS-CoV-2-infected patients have high clonal expansion (clone size≥2), especially in the SCoV13 subject, in which the proportion of clone size≥10 was even more than 70% (Figure 3(B)). IgA is the dominant isotype in the highly expanded clones (clone size≥10), followed by IgG in all groups (Figure 3(C)). Significant expansion of BCR clones in IgA/IgG/IgM isotypes was found in the SARS-CoV-2 infected group than that in other groups (Figure 3(D)). The top 1 clonal expansion antibody gene (IGHV3-33, IGHJ4, IGLV2-14, IGLJ3) was synthesized and subjected to antibody production in HEK293 cells. I40 exhibited high-affinity binding against SARS-CoV-2 S2 protein (Figure 3(E)). We found that the infected individuals show higher clonal expansion than the healthy and vaccinated healthy individuals. These results indicated that SARS-CoV-2 infection greatly enhanced BCR clonal expansion compared with the vaccinated by SARS-CoV-2 inactivated virus vaccine, especially in the IgA isotype.

### SARS-CoV-2 infection induced higher SHM compared with vaccination

**Figure 4.**
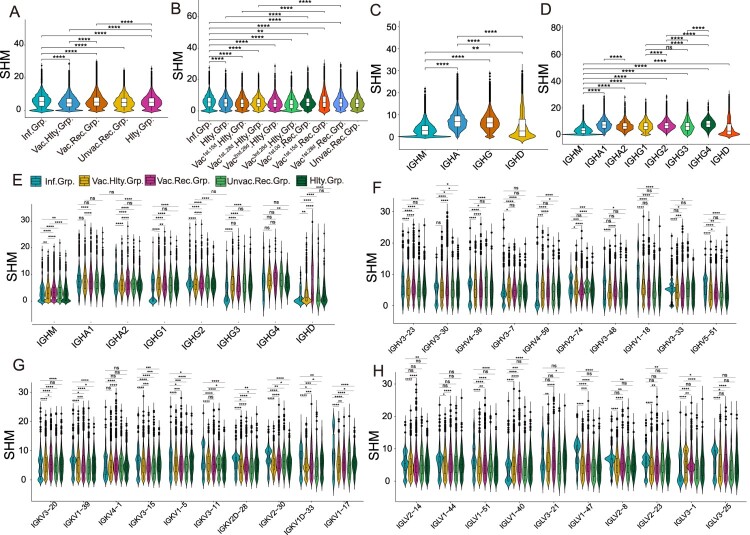
Characteristics of Somatic Hypermutation in Different Groups, Immunoglobulin isotypes and V genes. (A) The differential analysis of SHM rate in SARS-CoV-2 infected vaccinated healthy, vaccinated recovered, unvaccinated recovered and healthy groups. Statistical significance was evaluated using the Wilcoxon rank-sum test. Significance was defined as: *p-value < 0.05; **p-value < 0.01; ***p-value < 0.001 and **** *p*-value < 0.0001; not significant (ns): *p-*value > 0.05. (B) The differential analysis of SHM rate in SARS-CoV-2 infected, healthy, vaccinated^1st.15d^ healthy, vaccinated^1st.28d^ healthy, vaccinated^2nd.28d^ healthy, vaccinated^3rd.28d^ healthy, vaccinated^1st.0d^ recovered, vaccinated^1st.15d^ recovered, vaccinated^1st.28d^ recovered and unvaccinated recovered groups. Statistical significance was evaluated using the Wilcoxon rank-sum test. (C) The differential analysis of SHM rate in IgM, IgA, IgG and IgD. (D) The differential analysis of SHM rate in IgM, IgA1, IgA2, IgG1, IgG2, IgG3, IgG4 and IgD. (E) SHM rate of immunoglobulin isotypes subclass in the different groups. (F–H) SHM rate of immunoglobulin isotypes subclass in the heavy chain (F), kappa chain(G) and lambda chain (H). The 10 most common IGHVs/IGKVs/IGLVs are ordered by frequency in the patients.

SHM introduces point mutations in the antibody variable region that encodes the antigen-binding sites, thus promoting affinity maturation [20]. We next explored the difference in the SHM ratio in the above groups. SHM rate in the infected group was significantly higher than that in other groups (*p* < 0.05), and the vaccinated healthy group had the lowest level of SHM ratio (Figure 4(A)). We further investigated the SHM rate over time. In the vaccinated healthy and vaccinated recovered groups, the highest SHM rate was found in the vaccinated ^1st.15d^ recovered group (Figure 4(B)). For the SHM rate in various immunoglobulin isotypes, IgA accounted for the highest SHM rate and the lowest SHM rate appeared in IgM (*p* < 0.05, Figure 4(C,D)). Interestingly, the SHM rate in IgG1 or IgG3-expressing B cells of the infected group was prominently lower than that of other groups (Figure 4(E) and S3A).

Then we wondered whether the SHM rate of heavy, kappa and lambda chains differed between SARS-CoV-2 infection and vaccination. The top 10 heavy, kappa and lambda genes were selected to be analyzed, respectively (Figure 4(F, H) and S3B-D). The SHM rate of IGHV3-23, IGHV1-18 and IGHV5-51 in the SARS-CoV-2 infected and vaccinated recovered groups were higher than that in the vaccinated healthy and unvaccinated recovered groups (*p* < 0.05, Figure 4(F) and Figure S3B). The SHM rates of the top 10 light chain genes were different in the various groups (Figure 4(G,H) and S3C-D). Altogether, these results implied that higher SHM were induced by SARS-CoV-2 infection, with or without subsequent vaccination, compared with the vaccinated healthy group, although the SHM ratio is different in different isotypes.

### Paired public clonotypes are shared between SARS-CoV-2 infected and vaccinated groups

**Figure 5.**
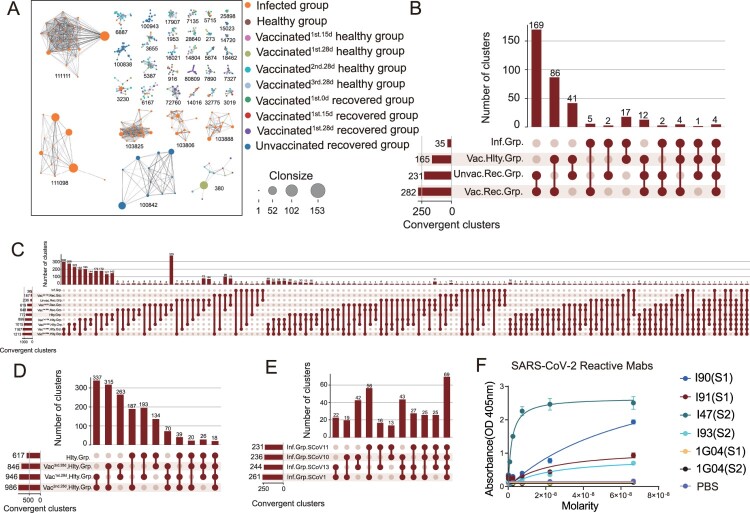
Convergent Paired Heavy and Light Sequences Shared between SARS-CoV-2 Infection and Vaccination. (A) The network diagram of clonotype cluster among in SARS-CoV-2 infected, healthy, vaccinated^1st.15d^ healthy, vaccinated^1st.28d^ healthy, vaccinated^2nd.28d^ healthy, vaccinated^3rd.28d^ healthy, vaccinated^1st.0d^ recovered, vaccinated^1st.15d^ recovered, vaccinated^1st.28d^ recovered and unvaccinated recovered groups. Only the nodes ≥10 clonotype clusters are shown in this figure. (B–E) Convergent paired heavy and light clusters among different groups (the top panel). The sample distribution is indicated by the lines and dots with the number of clusters sharing that group distribution indicated by the vertical histogram bars. The total number of convergent clusters identified in each group is indicated in the histogram to the left of the plot. (F) Binding of convergent antibodies to SARS-CoV-2 spike antigen by ELISA. Public antibody I47 was found among the infected, vaccinated recovered and vaccinated healthy subjects, I90 and I91 in the infected subjects, and I93 in the vaccinated healthy and vaccinated recovered subjects.

To investigate the potential convergent antibody response elicited by SARS-CoV-2 infection and vaccination, we performed a shared cluster analysis. Two definitions were used for the clonotype sharing: similar complementarity-determining region 3 (CDR3) amino acid (aa) sequence matches, or similar CDR3 aa sequence matches plus exact V and J gene usage. The python Scirpy package was used to calculate the BCR distance. The CDR3 aa alignment distance cut-off values of both heavy and light chains less than or equal to 10 were considered as a clonotype cluster. We first examined the clonotype cluster network diagram among the SARS-CoV-2 infected, vaccinated healthy, healthy, vaccinated recovered and unvaccinated recovered groups only based on CDR3 aa. A large number of clonotype clusters were shared in the above groups, such as clusters 380, 3230 and 6167 (only clonotype cluster nodes≥ 10 are shown in Figure 5(A) and S4A). Besides, clonotype clusters containing dominant clonotypes were identified in the infected and unvaccinated recovered groups. Several clonotypes with clone sizes of more than 20 were found in clusters 111111, 111098 and 100842 (Figure 5(A)). Interestingly, two BCR clonotypes (C5P4FS and C7P4FS) in cluster 100842 were SARS-CoV-2 RBD-reactive in our previous research [12]. The detailed information about clonotype clusters 380, 100842 and 111111 is shown in Table S1.

To understand the BCR repertoire evolution of SARS-CoV-2 infection and vaccination, we clustered CDR3 aa sequence matches plus exactly matched V and J gene usage, analyzing the convergent clusters (also named public antibody clonotypes) shared by the SARS-CoV-2 infected, vaccinated healthy, vaccinated recovered and unvaccinated recovered groups. Paired public antibody clonotypes were defined by sharing the same heavy chain (VH/JH) and light chain variable genes (VK/JK or VL/JL) and similar CDR3 amino acid sequences. Three hundred forty-three convergent clusters met the criterion in the SARS-CoV-2 infected, vaccinated healthy, vaccinated recovered and unvaccinated recovered groups, with 35 clusters in the infected group and 165 clusters in the vaccinated healthy group (Figure 5(B)). Ten convergent clusters spanned more than six groups (Figure 5(C)). The sequences of several multi-groups shared clonotype clusters (4, 758, 256 and 340) are shown in supplemental Table S2.

Next, we further explored the convergent clusters between the different time points following in the vaccinated healthy, vaccinated recovered and infected groups, respectively. The convergent clusters shared between vaccinated^1st28d^ healthy and vaccinated^2nd.28d^ healthy groups were the top 1 cluster in the vaccinated healthy group over time (Figure 5(D)). Seventy convergent clusters were found among the three shots of the SARS-CoV-2 inactivated virus vaccine (Figure 5(D)). Sixty-nine public clonotypes were spanning in the four infected subjects (Figure 5(E)). Besides, the convergent clusters shared between the vaccinated recovered and unvaccinated recovered groups, and the infected and vaccinated healthy groups are present in Figure S4B,C, respectively. The vaccinated recovered group generated a higher number of convergent clusters than the healthy subjects with BBIBP-CorV vaccination (421 VS 237) at day 0 and day 28 (Figure S4D,E). To identify the antigen specificity of the convergent clonotypes with paired heavy and light chains, we generated several convergent mAbs from the HEK293 cells. Four public antibodies from different individuals (infected, vaccinated healthy and vaccinated recovered groups) were confirmed as SARS-CoV-2 spike-specific (Figure 5(F)).

Briefly, a small proportion of convergent clusters were shared among the SARS-CoV-2 infected, vaccinated healthy, vaccinated recovered and unvaccinated recovered groups, while numerous convergent clusters were found across the SARS-CoV-2 infected patients.

### Characteristics of CDRH3 in SARS-CoV-2 infection and vaccination

**Figure 6.**
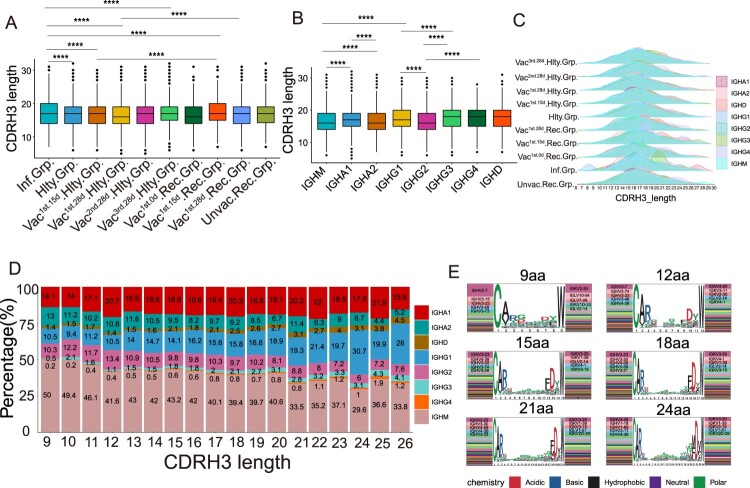
Characteristics of CDRH3 in SARS-CoV-2 Infection and Vaccination. (A) The differential analysis of CDRH3 length in SARS-CoV-2 infected, healthy, vaccinated^1st.15d^ healthy, vaccinated^1st.28d^ healthy, vaccinated^2nd.28d^ healthy, vaccinated^3rd.28d^ healthy, vaccinated^1st.0d^ recovered, vaccinated^1st.15d^ recovered, vaccinated^1st.28d^ recovered and unvaccinated recovered groups. Statistical significance was evaluated using the two-sided *t*-test. Significance was defined as: *p-value < 0.05; **p-value < 0.01; ***p-value < 0.001 and **** *p*-value < 0.0001; not significant (ns): *p*-value > 0.05. (B) The differential analysis of CDRH3 length in IgM, IgA1, IgA2, IgG1, IgG2, IgG3, IgG4 and IgD. Statistical significance was evaluated using the two-sided t­-test. (C) Distribution of CDR3 length of each group in different immunoglobulin isotypes. (D) The Bar plot shows the proportion of immunoglobulin isotypes in different CDRH3 lengths, including IgM, IgA1, IgA2, IgG1, IgG2, IgG3, IgG4 and IgD. (E) The heavy and light genes usage, and CDR3 motif of clonal BCR which CDR3 length is 9aa, 12aa, 15aa, 18aa, 21aa and 24aa.

We explored the characteristics of CDRH3 sequences of clonal expended BCR in SARS-CoV-2 infection and vaccination. The length of CDRH3 in the SARS-CoV-2 infected and vaccinated^1st.15d^ recovered groups was notably longer than that in the vaccinated healthy, unvaccinated recovered and healthy groups (*p* < 0.05, Figure 6(A)). The length of CDRH3 length varied across different immunoglobulin isotypes. IgM had the shortest CDRH3 length in all the immunoglobulin isotypes (*p* < 0.05, Figure 6(B)). The immunoglobulin isotypes in the infected group had a greater diversity of the CDRH3 length than that in the vaccinated healthy group, especially for the IgG3 subclass (Figure 6(C)). Noteworthy, the strongest negative correlations were seen between CDRH3 length and IgM proportion (*r* = −0.915, *p* < 0.0001), and IgA2 proportion (*r* = −0.771, *p* < 0.0001), especially in vaccinated healthy and vaccinated recovered groups (Figure 6(D) and S5A–D). There was the strongest positive correlation between CDRH3 length and IgG1 proportion (*r* = 0.967, *p* < 0.0001), and IgD proportion (*r* = 0.950, *p* < 0.0001), especially in the vaccinated recovered group. However, there was no correlation between CDRH3 length and IgA1 (Figure 6(D)). The short length of CDRH3 preferred to use IGHV3-7/IGKV2-30 or IGHV3-7/IGKV3-20, which in the long length CDR3 were IGHV3-23/IGKV3-20 or IGHV3-30/IGKV3-20, suggesting that the VH/VK genes bias usage in different length of CDRH3 (Figure 6(E)). Immunoglobulin isotypes and CDRH3 length between the infected individuals and vaccinated individuals were different at distinct time points (Figure S5E,F). However, no obvious differences were observed among the vaccinated individuals at different time points. In summary, severe SARS-CoV-2 infection was likely to induce longer CDRH3. A strong correlation between CDRH3 length and IgG1, IgA2, and IgM was found in the vaccinated healthy and vaccinated recovered groups, but not after SARS-CoV-2 infection alone.

## Discussion

Clarifying signatures of single-cell paired heavy and light chain BCR repertoire of SARS-CoV-2 infection and vaccination can help expose the nature of COVID-19 and guide therapeutic agent development as well as vaccine design and assessment [7,21]. We compared the global paired BCR repertoire signature induced by SARS-CoV-2 infection and vaccination via a single-cell V(D)J sequence. Our results revealed preferential V genes usage was more prominent in the SARS-CoV-2 infected group than that in the vaccinated healthy group. Natural infection enhanced BCR clonal expansion and SHM compared with the vaccinated SARS-CoV-2-inactivated virus vaccine. Interestingly, a small proportion of public clonotypes was shared by the SARS-CoV-2-infected, vaccinated healthy, vaccinated recovered and unvaccinated recovered groups.

Increasing our understanding of the paired BCR repertoire in the context of COVID-19 is essential given its role in defense against SARS-CoV-2 infection, and potentially in preventing secondary infection and re-infection [8,10,22,23]. IGHV genes covered a majority of pathogen-engaging regions of BCR, including the three complementarity-determining regions CDRH1, CDRH2, and a portion of CDRH3 [7]. Therefore, we investigated whether there are any differences in V gene usage between SARS-CoV-2 infection and vaccination, which may indicate preferences relevant for response to a particular pathogen. Our analysis revealed higher frequency changes in the paired IGHV and IGLV of BCRs in SARS-CoV-2 infected compared with the vaccinated healthy group. We found the preferential V gene segment usage of BCR was more prominent in the SARS-COV-2-infected group than that in the vaccinated healthy group. IGHV3-33/IGLV2-14 was the top paired BCR clonotype in the infected group, while IGHV3-23/IGKV3-20 was the dominant paired clonotype in the vaccinated healthy group. SARS-CoV-2 infection greatly enhanced BCR clonal expansion in our study, it was the same as previous studies [24,25], indicating that B cell activity and humoral immune responses were strongly activated in infected patients [25].

A higher SHM rate was observed in the SARS-CoV-2-infected group compared to the vaccinated recovered group, which may be due to the B cells continuously being exposed to SARS-CoV-2 antigen during infection in the human body (Figure 4(A)). Naturally, infection often has a larger dose of the antigen and longer time of antigen exposure than vaccination. These antigens can make the B cells undergo SHM and affinity maturation multiple times, causing higher antibody response [11]. The vaccinated^1st.15d^ recovered group has the highest SHM rate among all the groups at different time points, but the SHM rate decreases sharply at 28 days post-vaccination (Figure 4(B)). It indicated that the exposure to SARS-CoV-2 antigen cannot last 28 days post-vaccination and likely cannot drive B cells SHM anymore. Moreover, the vaccinated recovered group has high SHM rates is likely due to more prominent memory B cell reactivation than the vaccinated healthy group, due to previous SARS-CoV-2 exposure. Previously infected SARS-CoV-2 subjects following vaccination with mRNA BNT162b2, mRNA-1273 and Ad26.COV2.S can induce potent and broad neutralizing antibody responses [26–30]. If memory B cell responses evolve similarly in naïve individuals who receive vaccines, additional appropriately timed boosting with available vaccines should lead to protective immunity against circulating Variants of Concern (VOCs) [31].

Despite the diversity of antigen-driven antibody responses, many researchers have previously identified patterns of highly similar, “convergent” antibodies shared by different individuals in response to pathogens, such as EBOV [32,33], dengue virus [34], influenza virus [35] and HIV [36]. Similar phenomena have been observed in patients infected by SARS-CoV-2 [2,37–39]. Some convergent B cell clonotypes presented cross-reactive against the SARS-CoV and SARS-CoV-2 RBD [2]. Zhang reported that 21.7% (168/774) published monoclonal antibodies (mAbs) were found to have the same IGHV genes and at least 80% CDRH3 aa sequence identity compared to the BCR repertoires from COVID-19 recovered patients. Thirty-six percent (61/168) of them showed neutralizing ability against SARS-CoV-2 [20]. Chen found that heavy chain germline-revertant forms of public antibody clonotypes bind efficiently to spike protein [40]. In our study, we approved that paired heavy and light chain public clonotype antibodies from different individuals after infection or vaccination were SARS-CoV-2 spike-specific. These results supported that SARS-CoV-2 induced convergent antibody response across different patients, which can be identified by querying antibody sequencing repertoires if enough documented antigen-specific convergent clonotypes are known.

As the SARS-CoV-2 epidemic continues, the exposure history of the population to the virus is complicated. Varying immune exposure history requires different SARS-CoV-2 vaccination strategies. Learning the dynamics and characteristics of the paired heavy and light chain BCR repertoires following SARS-CoV-2 infection and vaccination can lead us to understand how the humoral immune response under different immune exposure histories. We found that BCR variable (V) genes were more prominently used in the SARS-CoV-2 infected group than in the vaccinated healthy group. SARS-CoV-2 infection enhanced BCR clonal expansion and more somatic hypermutation than the vaccinated healthy group.

Natural infection almost always caused better immunity than vaccines [41]. The results indicated that we can monitor the vaccine efficacy by identifying the rate of BCR clonal expansion and somatic hypermutation. Public clonotypes were shared by the SARS-CoV-2 infected, vaccinated healthy and vaccinated recovered individuals and some of them were SARS-CoV-2 spike-specific. Our study offered a comprehensive characterization of the paired BCR repertoire in the varying nature of SARS-CoV-2 exposure. It would potentially inform the design of germline-targeting SARS-CoV-2 antigen, to efficiently prime specific protective B cells’ immune response with special enriched types of VH genes or with antigen-reactive public clonotypes VH genes.

## Supplementary Material

Supplemental Material

## Data Availability

The raw sequencing data have been deposited into the Genome Sequence Archive of National Genomics Data Center (CNCB), under accession code HRA002583 at https://ngdc.cncb.ac.cn/gsa-human/. The authors declare that the code used for the current study is available from the lead contact (Prof. Yao-Qing Chen, chenyaoqing@mail.sysu.edu.cn) upon request.
