# Supplementary material for: Comparative global B cell receptor repertoire difference induced by SARS-CoV-2 infection or vaccination via single-cell V(D)J sequencing
Source: Emerg Microbes Infect. 2022 Aug 11;11(1):2007–20. doi: 10.1080/22221751.2022.2105261 (PMC9377262; doi:10.1080/22221751.2022.2105261)
Supplement: Supplemental Material [file TEMI_A_2105261_SM7329.docx]

**Figure 1.** Preferential V Gene Segments Usage of BCR in SARS-CoV-2 Infection and Vaccination.

1. Schematic overview of the 10X Genomics single-cell V(D)J sequencing process. The global BCR repertoire characterization in different nature of SARS-CoV-2 exposure.
2. The distribution of top 5 paired heavy and light chain in SARS-CoV-2 infected (Inf.Grp.), healthy (Hlty.Grp.), vaccinated healthy (Vac.Hlty.Grp.), vaccinated recovered (Vac.Rec.Grp.) and unvaccinated recovered groups (Unvac. Rec.Grp.).
3. Sankey diagram shows total BCR specific heavy and light VJ pairs that occurred more than once.

**(D-G)** The frequency and differential analysis of VH gene segments between healthy and infected groups**(D)**, healthy and vaccinated healthy groups **(E)**, vaccinated healthy and infected groups **(F)**, unvaccinated recovered and vaccinated recovered groups **(G)**. The colors represent the p-value of the significant positive or negative. Red: p<0.05, gray: p>0.05. Fisher’s exact test, p-value less than 0.05, was considered to be statistically significant.

**(H)** Polar plot shows the relative changes of VH genes usage in SARS-CoV-2 infected (Inf.Grp), vaccinated^1st.15d^ healthy (Vac^1st.15d^.Hlty.Grp.) vaccinated^1st.28d^ healthy (Vac^1st.28d^.Hlty.Grp.),vaccinated^2nd.28d^ healthy (Vac^2nd.28d^.Hlty.Grp.)and vaccinated^3rd.28d^ healthy(Vac^3rd.28d^.Hlty.Grp.) groups. The VH gene usage proportion of healthy group (Hlty.Grp.) was considered as the baseline. The relative changes of VH genes usage were calculated by the following method: the proportion of VH gene at a certain time point minus above baseline.

**(I)** Polar plot shows the relative changes of VH genes usage in vaccinated^1st.0d^ recovered (Vac^1st.0d^.Rec.Grp.), vaccinated^1st.15d^ recovered (Vac^1st.15d^.Rec.Grp.), vaccinated^1st.28d^ recovered(Vac^1st.28d^.Rec.Grp.) and unvaccinated recovered (Unvac.Rec.Grp.) groups. The calculation method of relative changes of VH genes usage was same as figure 1(H).

**Figure 2.** Immunoglobulin Isotypes Signature Analysis of SARS-CoV-2 Infection and Vaccination.

1. The bar graph shows immunoglobulin isotypes distribution in Total, SARS-CoV-2 infected, vaccinated healthy, vaccinated recovered, unvaccinated recovered and healthy groups.
2. The bar graph shows immunoglobulin isotypes distribution in SARS-CoV-2 infected, healthy, vaccinated^1st.15d^ healthy, vaccinated^1st.28d^ healthy, vaccinated^2nd.28d^ healthy, vaccinated^3rd.28d^ healthy, vaccinated^1st.0d^ recovered, vaccinated^1st.15d^ recovered, vaccinated^1st.28d^ recovered and unvaccinated recovered groups.

**(C-E)** The frequency and differential analysis of immunoglobulin isotypes between vaccinated healthy and infected groups**(C)**, vaccinated healthy and unvaccinated recovered groups**(D)**, healthy and vaccinated healthy groups**(E)**. The colors represent the p-value of the significant positive or negative, red: p<0.05, grey: p>0.05. Fisher’s exact test, p-value less than 0.05, was considered to be statistically significant.

**(F)** Combination of V genes of heavy chain and light chain. The combination of V genes of heavy chain and light chain of IgA and IgG in infected, vaccinated healthy and vaccinated recovered groups.

**Figure 3.** BCR Clonal Expansion Following SARS-CoV-2 Infection and Vaccination.

1. Bar plot shows clonal expansion distribution in SARS-CoV-2 infected, healthy, vaccinated^1st.15d^ healthy, vaccinated^1st.28d^ healthy, vaccinated^2nd.28d^ healthy, vaccinated^3rd.28d^ healthy, vaccinated^1st.0d^ recovered, vaccinated^1st.15d^ recovered, vaccinated^1st.28d^ recovered and unvaccinated recovered groups.The different colors represent the clone size.
2. Bar plot shows the clonal expansion distribution in each individual sample.
3. Bar plot shows clonal expansion distribution in different immunoglobulin isotypes, including IgM, IgA1, IgA2, IgG1, IgG2, IgG3, IgG4 and IgD.
4. Bar plot shows clonal expansion distribution of different immunoglobulin isotypes in different groups.

**(E)**  Binding of top1 dominant antibody to SARS-CoV-2 S2 antigen by ELISA.

**Figure 4.** Characteristics of Somatic Hypermutation in Different Groups, Immunoglobulin isotypes and V genes.

**(A)** The differential analysis of SHM rate in SARS-CoV-2 infected, vaccinated healthy, vaccinated recovered, unvaccinated recovered and healthy groups. Statistical significance was evaluated using the Wilcoxon rank-sum test. Significance was defined as: *p value < 0.05; **p value < 0.01; ***p value < 0.001 and **** p value < 0.0001; not significant (ns): p value > 0.05.

**(B)** The differential analysis of SHM rate in SARS-CoV-2 infected, healthy, vaccinated^1st.15d^ healthy, vaccinated^1st.28d^ healthy, vaccinated^2nd.28d^ healthy, vaccinated^3rd.28d^ healthy, vaccinated^1st.0d^ recovered, vaccinated^1st.15d^ recovered, vaccinated^1st.28d^ recovered and unvaccinated recovered groups. Statistical significance was evaluated using the Wilcoxon rank-sum test.

**(C)** The differential analysis of SHM rate in IgM, IgA, IgG and IgD.

**(D)** The differential analysis of SHM rate in IgM, IgA1, IgA2, IgG1, IgG2, IgG3, IgG4 and IgD.

**(E)** SHM rate of immunoglobulin isotypes subclass in the different groups.

**(F-H)** SHM rate of immunoglobulin isotypes subclass in heavy chain **(F)**, kappa chain**(G)** and lambda chain**(H)**. The 10 most common IGHVs/IGKVs/IGLVs are ordered by frequency in the patients.

**Figure 5.** Convergent Paired Heavy and Light Sequences Shared between SARS-CoV-2 Infection and Vaccination.

1. The network diagram of clonotype cluster among in SARS-CoV-2 infected, healthy, vaccinated^1st.15d^ healthy, vaccinated^1st.28d^ healthy, vaccinated^2nd.28d^ healthy, vaccinated^3rd.28d^ healthy, vaccinated^1st.0d^ recovered, vaccinated^1st.15d^ recovered, vaccinated^1st.28d^ recovered and unvaccinated recovered groups. Only the nodes ≥10 clonotype clusters were shown in this figure.

**(B-E)** Convergent paired heavy and light clusters among different groups (top panel). The sample distribution is indicated by the lines and dots with the number of clusters sharing that group distribution indicated by the vertical histogram bars. The total number of convergent clusters identified in each group is indicated in the histogram to the left of the plot.

**(F)** Binding of convergent antibodies to SARS-CoV-2 spike antigen by ELISA. Public antibody I47 was found between the infected, vaccinated recovered and vaccinated healthy subjects, I90 and I91 in the infected subjects, and I93 in the vaccinated healthy and vaccinated recovered subjects.

**Figure 6.** Characteristics of CDRH3 in SARS-CoV-2 Infection and Vaccination.

**(A)** The differential analysis of CDRH3 length in SARS-CoV-2 infected, healthy, vaccinated^1st.15d^ healthy, vaccinated^1st.28d^ healthy, vaccinated^2nd.28d^ healthy, vaccinated^3rd.28d^ healthy, vaccinated^1st.0d^ recovered, vaccinated^1st.15d^ recovered, vaccinated^1st.28d^ recovered and unvaccinated recovered groups. Statistical significance was evaluated using the two-sided t test. Significance was defined as: *p value < 0.05; **p value < 0.01; ***p value < 0.001 and **** p value < 0.0001; not significant (ns): p value > 0.05.

**(B)** The differential analysis of CDRH3 length in IgM, IgA1, IgA2, IgG1, IgG2, IgG3, IgG4 and IgD. Statistical significance was evaluated using the two-sided t test.

**(C)** Distribution of CDR3 length of each group in different immunoglobulin isotypes.

**(D)** Bar plot shows the proportion of immunoglobulin isotypes in different CDRH3 length, including IgM, IgA1, IgA2, IgG1, IgG2, IgG3, IgG4 and IgD.

**(E)** The heavy and light genes usage, and CDR3 motif of clonal BCR which CDR3 length is 9aa, 12aa, 15aa, 18aa, 21aa and 24aa.

**Supplementary materials**

Supplementary Figure 1. Sorting strategy of memory and plasma B cells.

Supplementary Figure 2. Preferential VK/VL gene segments usage in SARS-CoV-2 infection and vaccination.

(A) Polar plot shows the relative changes of VK genes usage in SARS-CoV-2 infected, vaccinated^1st.15d^ healthy, vaccinated^1st.28d^ healthy, vaccinated^2nd.28d^ healthy and vaccinated^3rd.28d^ healthy groups.

(B) Radar chart shows the relative changes of VK genes usage in vaccinated^1st.0d^ recovered, vaccinated^1st.15d^ recovered, vaccinated^1st.28d^ recovered unvaccinated recovered groups.

(C) Polar plot shows the relative changes of VL genes usage in SARS-CoV-2 infected, vaccinated^1st.15d^ healthy, vaccinated^1st.28d^ healthy, vaccinated^2nd.28d^ healthy and vaccinated^3rd.28d^ healthy groups.

(D) Polar plot shows the relative changes of VL genes usage in vaccinated^1st.0d^ recovered, vaccinated^1st.15d^ recovered, vaccinated^1st.28d^ recovered unvaccinated recovered groups.

Supplementary Figure 3. The difference of SHM rate in immunoglobulin isotypes and the top 10 V genes.

(A) The differential analysis of SHM rate of immunoglobulin isotypes in different groups. (B)The differential analysis of SHM rate of the top 10 heavy chain in different groups. (C)The differential analysis of SHM rate of the top 10 kappa chain. (D)The differential analysis of SHM rate of the top 10 lambda chain.

Supplementary Figure 4. Convergent paired heavy and light sequences shared between different groups.

(A)The network diagram of clonotype cluster among in infected, vaccinated healthy, vaccinated recovered, unvaccinated recovered and healthy groups. Only the nodes ≥10 clonotype clusters were shown in this figure.

(B-D) Convergent paired heavy and light clusters among patient groups (top panel). The groups distribution is indicated by the lines and dots with the number of clusters sharing that sample distribution indicated by the vertical histogram bars. The total number of convergent clusters identified in each sample is indicated in the histogram to the left of the plot.

Supplementary Figure 5. The Proportion of Immunoglobulin Isotypes in Different CDRH3 Length

Bar plot shows the proportion of immunoglobulin isotypes in different CDRH3 length according to SARS-CoV-2 infected group (A), vaccinated healthy group (B), vaccinated recovered group(C) and unvaccinated recovered group(D). Principal component analysis (PCA) for isotypes distribution among individuals at different timepoints(E). Principal component analysis (PCA) for CDRH3 length distribution among individuals at different timepoints(F).

Table S1. The basic information of Vaccinated Healthy group

| Subject | Sex | Age (years) | Date of first dose BBIBP-CorV | Date of second dose BBIBP-CorV | Date of third dose BBIBP-CorV |
| --- | --- | --- | --- | --- | --- |
| SD01 | Male | 25 | April 21, 2021 | May 20, 2021 | November 26, 2021 |
| SD05 | Female | 23 | April 21, 2021 | May 20, 2021 | November 26, 2021 |
| SD10 | Male | 28 | April 21, 2021 | May 20, 2021 | November 26, 2021 |
| SD13 | Female | 24 | April 21, 2021 | May 20, 2021 | November 26, 2021 |
| SD14 | Female | 24 | April 21, 2021 | May 20, 2021 | November 26, 2021 |
| SD15 | Female | 30 | April 21, 2021 | May 20, 2021 | November 26, 2021 |

Table S2. The basic information of Vaccinated Recovered group

| Subject | Sex | Age (years) | Date of discharged | Date of PBMC collection (0d) | Date of PBMC collection (15d) | Date of PBMC collection (28d) |
| --- | --- | --- | --- | --- | --- | --- |
| KD01 | Male | 63 | March 30, 2020 | April 29, 2021 | May 14, 2021 | May 28, 2021 |
| KD02 | Female | 62 | March 30, 2020 | April 29, 2021 | May 14, 2021 | May 28, 2021 |
| KD10 | Male | 43 | March 15, 2020 | September 16, 2021 | September 30, 2021 | October 14, 2021 |
| KD14 | Female | 37 | February 23,2020 | September 30, 2021 | October 14, 2021 | October 14, 2021 |
| KD15 | Male | 36 | February 17,2020 | September 30, 2021 | October 14, 2021 | October 14, 2021 |

Table S3. The basic information of Unvaccinated Recovered group

| Subjects | Sex | Age (years) | Symptom | Date of symptom onset | Discharged | Date of PBMC collection | Variants of SARS-CoV-2 |
| --- | --- | --- | --- | --- | --- | --- | --- |
| CV1 | Male | 29 | severe | January 27, 2020 | February 20, 2020 | February 28, 2020 | Wild type |
| CV2 | Female | 35 | mild | January 31, 2020 | February 23, 2020 | Marth 7, 2020 | Wild type |
| CV3 | Male | 56 | mild | February 3, 2020 | February 25, 2020 | February 25, 2020 | Wild type |
| CV4 | Female | 59 | mild | January 16, 2020 | February 3, 2020 | February 25, 2020 | Wild type |
| CV5 | Female | 47 | severe | January 24, 2020 | February 22, 2020 | Marth 7, 2020 | Wild type |

Table S4. The basic information of Infected group

| Subjects | Sex | Age(years) | Medical history | Symptom | Time of blood sampling (days post symptoms onset) |
| --- | --- | --- | --- | --- | --- |
| SCoV1-M0 | Male | 60 | Obesity | severe | 14 |
| SCoV10-M0 | Female | 59 | None | severe | 11 |
| SCoV11-M0 | Male | 57 | Type II diabetes | severe | 33 |
| SCoV13-M0 | Male | 51 | None | severe | 24 |

Table S5. The detailed information of clonotype cluster 380,100842 and 111111 in Figure 5A

| **clonotype cluster id** | **IGHV** | **IGHJ** | **CDRH3** | **IGK/LV** | **IGK/LJ** | **CDRK/L3** | **sample** |
| --- | --- | --- | --- | --- | --- | --- | --- |
| 380 | IGHV3-7 | IGHJ4 | CVRGSIDSW | IGLV10-54 | IGLJ3 | CSAWDSSLSAWVF | Hlty.Grp. |
| 380 | IGHV3-7 | IGHJ4 | CARGSIDYW | IGLV10-54 | IGLJ3 | CSAWDTSLSAWVF | Hlty.Grp. |
| 380 | IGHV3-7 | IGHJ2 | CARGDLDLW | IGLV10-54 | IGLJ3 | CSAWDISLGAWVF | Vac.Hlty.Grp. |
| 380 | IGHV3-7 | IGHJ4 | CVTGDVDYW | IGLV10-54 | IGLJ3 | CSAWDSSLRAWVF | Vac.Hlty.Grp. |
| 380 | IGHV3-7 | IGHJ4 | CATGDFDYW | IGLV10-54 | IGLJ3 | CSAWDSSLTAWVF | Vac.Hlty.Grp. |
| 380 | IGHV3-7 | IGHJ4 | CVRGSIDYW | IGLV10-54 | IGLJ3 | CSAWDSSLSAWVF | Vac.Hlty.Grp. |
| 380 | IGHV3-48 | IGHJ4 | CARGTLDFW | IGLV10-54 | IGLJ3 | CSAWDNNLSAWVF | Vac.Rec.Grp. |
| 380 | IGHV3-7 | IGHJ2 | CARGDLDLW | IGLV10-54 | IGLJ3 | CSAWDTSLGAWVF | Vac.Hlty.Grp. |
| 380 | IGHV1-46 | IGHJ4 | CARGDIDYW | IGLV10-54 | IGLJ3 | CSAWDSSLSAWVF | Vac.Hlty.Grp. |
| 380 | IGHV3-7 | IGHJ4 | CARGDFDYW | IGLV10-54 | IGLJ3 | CSAWDSSLSAWVF | Vac.Hlty.Grp. |
| 380 | IGHV1-46 | IGHJ4 | CARGDIDYW | IGLV10-54 | IGLJ3 | CSAWDSSLSAWVF | Vac.Rec.Grp. |
| 380 | IGHV3-7 | IGHJ4 | CGTGDFDFW | IGLV10-54 | IGLJ3 | CSAWDISLSAWVF | Vac.Rec.Grp. |
| 100842 | IGHV3-74 | IGHJ3 | CARERPPDLRWWGSEPSEPQQGGGFDMW | IGKV3-11 | IGKJ4 | CQQRSNWPLTF | Unvac.Rec.Grp. |
| 100842 | IGHV3-74 | IGHJ3 | CARERPPDLRWWGSEPSEPQQGSGFDLW | IGKV3-11 | IGKJ4 | CQQRSNWPLTF | Unvac.Rec.Grp. |
| 100842 | IGHV3-74 | IGHJ3 | CTRERPPDLRWWGSEPSEPQQGGGFDVW | IGKV3-11 | IGKJ4 | CQQRSNWPLTF | Unvac.Rec.Grp. |
| 100842 | IGHV3-74 | IGHJ3 | CARERPPDLRWWGSEPSEPQQGSGFDLW | IGKV3-11 | IGKJ4 | CQQRSNWPLTF | Unvac.Rec.Grp. |
| 100842 | IGHV3-74 | IGHJ3 | CTRERPPDLRWWGSEPSEPQQGGGFDVW | IGKV3-11 | IGKJ4 | CQQRSNWPLTF | Unvac.Rec.Grp. |
| 100842 | IGHV3-74 | IGHJ3 | CARERPPDLRWWGSEPSEPQQGGGFDIW | IGKV3-11 | IGKJ4 | CQQRSNWPLTF | Unvac.Rec.Grp. |
| 100842 | IGHV3-74 | IGHJ3 | CARERPPDLRWWGSEPSEPQQGGGFDIW | IGKV3-11 | IGKJ4 | CQQRSNWPLTF | Unvac.Rec.Grp. |
| 100842 | IGHV3-74 | IGHJ3 | CTRERPPDLRWWGSEPSEPQQGGGFDIW | IGKV3-11 | IGKJ4 | CQQRSNWPLTF | Unvac.Rec.Grp. |
| 100842 | IGHV3-74 | IGHJ3 | CTRERPPDLRWWGSEPSEPQQGGGFDVW | IGKV3-11 | IGKJ4 | CQQRSNWPLTF | Unvac.Rec.Grp. |
| 100842 | IGHV3-74 | IGHJ3 | CTRERPPDLRWWGSEPSEPQQGGGFDVW | IGKV3-11 | IGKJ4 | CQQRSDWPLTF | Unvac.Rec.Grp. |
| 100842 | IGHV3-74 | IGHJ3 | CTRERPPDLRWWGSEPSEPQQGGAFDVW | IGKV3-11 | IGKJ4 | CQQRSNWPLTF | Unvac.Rec.Grp. |
| 100842 | IGHV3-74 | IGHJ3 | CTRERPPDLRWWGNEPSEPQQGGGFDVW | IGKV3-11 | IGKJ4 | CQQRSNWPLTF | Unvac.Rec.Grp. |
| 100842 | IGHV3-74 | IGHJ3 | CARERPPDLRWWGSEPSEPQQGSGFDLW | IGKV3-11 | IGKJ4 | CQQRSSWPLTF | Unvac.Rec.Grp. |
| 100842 | IGHV3-74 | IGHJ3 | CARERPPDLRWWGSEPSEPQQGGGFDVW | IGKV3-11 | IGKJ4 | CQQRSNWPLTF | Unvac.Rec.Grp. |
| 100842 | IGHV3-74 | IGHJ3 | CTRERPPDLRWWGSEPSEPQQGGGFDVW | IGKV3-11 | IGKJ4 | CQQRSNWPLTF | Unvac.Rec.Grp. |
| 100842 | IGHV3-74 | IGHJ3 | CTRERPPDLRWWGSEPSEPQQGGGFDVW | IGKV3-11 | IGKJ4 | CQQRNNWPLTF | Unvac.Rec.Grp. |
| 111111 | IGHV6-1 | IGHJ3 | CARGWGMVRSGWPTDLFDIW | IGKV3-20 | IGKJ1 | CQQYGTSPTF | Inf.Grp. |
| 111111 | IGHV6-1 | IGHJ3 | CARAWGMVRSGWPTDLFDIW | IGKV3-20 | IGKJ1 | CQQYGTSPTF | Inf.Grp. |
| 111111 | IGHV6-1 | IGHJ3 | CARAWGMVRSGWPTDLFDIW | IGKV3-20 | IGKJ1 | CQQYGTSPTF | Inf.Grp. |
| 111111 | IGHV6-1 | IGHJ3 | CARGWGMVRSGWPTDLFDIW | IGKV3-20 | IGKJ1 | CQQYGSSPTF | Inf.Grp. |
| 111111 | IGHV6-1 | IGHJ3 | CARNWGMVRSGWPTDLFDIW | IGKV3-20 | IGKJ1 | CQQYGTSPTF | Inf.Grp. |
| 111111 | IGHV6-1 | IGHJ3 | CSRSWGMVRSGWPTDLFDIW | IGKV3-20 | IGKJ1 | CQQYGTSPTF | Inf.Grp. |
| 111111 | IGHV6-1 | IGHJ3 | CARGWGMVRSGWPTDLFDIW | IGKV3-20 | IGKJ1 | CQQYGTSPTF | Inf.Grp. |
| 111111 | IGHV6-1 | IGHJ3 | CARSWGMVRSGWPTDLFDFW | IGKV3-20 | IGKJ1 | CQQYGTSPTF | Inf.Grp. |
| 111111 | IGHV6-1 | IGHJ3 | CARGWGMVRSGWPTDLFDIW | IGKV3-20 | IGKJ1 | CQQYGTSPTF | Inf.Grp. |
| 111111 | IGHV6-1 | IGHJ3 | CARAWGVVRSGWPTDLFDIW | IGKV3-20 | IGKJ1 | CQQYGTSPTF | Inf.Grp. |
| 111111 | IGHV6-1 | IGHJ3 | CARGWGMVRSGWPTDLFDLW | IGKV3-20 | IGKJ1 | CQQYGTSPTF | Inf.Grp. |
| 111111 | IGHV6-1 | IGHJ3 | CARGWGMVRSGWPTDLFDIW | IGKV3-20 | IGKJ1 | CQQYGTSPTF | Inf.Grp. |
| 111111 | IGHV6-1 | IGHJ3 | CARGWGMVRSGWPTDLFDMW | IGKV3-20 | IGKJ1 | CQQYGTSPTF | Inf.Grp. |
| 111111 | IGHV6-1 | IGHJ3 | CARGWGMVRSGWPTDLFDIW | IGKV3-20 | IGKJ1 | CQQYGTSPTF | Inf.Grp. |
| 111111 | IGHV6-1 | IGHJ3 | CARAWGMVRSGWPTDLFDIW | IGKV3-20 | IGKJ1 | CQQYGTSPTF | Inf.Grp. |
| 111111 | IGHV6-1 | IGHJ3 | CSRSWGMVRSGWPTDLFDIW | IGKV3-20 | IGKJ1 | CQQYGTSPTF | Inf.Grp. |
| 111111 | IGHV6-1 | IGHJ3 | CARGWGMVRSGWPTDLFDIW | IGKV3-20 | IGKJ1 | CQQYGTSPTF | Inf.Grp. |
| 111111 | IGHV6-1 | IGHJ3 | CARGWGVVRSGWPTDLFDIW | IGKV3-20 | IGKJ1 | CQQYGTSPTF | Inf.Grp. |
| 111111 | IGHV6-1 | IGHJ3 | CARGWGMVRSGWPTDLFDIW | IGKV3-20 | IGKJ1 | CQQYGTSPTF | Inf.Grp. |
| 111111 | IGHV6-1 | IGHJ3 | CARGWGMVRSGWPTDLFDVW | IGKV3-20 | IGKJ1 | CQQYGSSPTF | Inf.Grp. |
| 111111 | IGHV6-1 | IGHJ3 | CSRGWGMVKSGWPTDLFDIW | IGKV3-20 | IGKJ1 | CQQYGTSPTF | Inf.Grp. |
| 111111 | IGHV6-1 | IGHJ3 | CARGWGMVRSGWPTDLFDIW | IGKV3-20 | IGKJ1 | CQQYGTSPTF | Inf.Grp. |
| 111111 | IGHV6-1 | IGHJ3 | CARAWGMVRSGWPTDLFDIW | IGKV3-20 | IGKJ1 | CQQYGTSPTF | Inf.Grp. |
| 111111 | IGHV6-1 | IGHJ3 | CARAWGMVRSGWPTDLFDIW | IGKV3-20 | IGKJ1 | CQQYVTSPTF | Inf.Grp. |
| 111111 | IGHV6-1 | IGHJ3 | CARSWGMVRSGWPTDLFDIW | IGKV3-20 | IGKJ1 | CQQYGTSPTF | Inf.Grp. |
| 111111 | IGHV6-1 | IGHJ3 | CARGWGMVRSGWPTDLFDIW | IGKV3-20 | IGKJ1 | CQQYGTSPTF | Inf.Grp. |
| 111111 | IGHV6-1 | IGHJ3 | CARGWGMVRSGWPTDLFDIW | IGKV3-20 | IGKJ1 | CQQYGTSPTF | Inf.Grp. |
| 111111 | IGHV6-1 | IGHJ3 | CARGWGMVRSGWPTDLFDMW | IGKV3-20 | IGKJ1 | CQQYGSSPTF | Inf.Grp. |
| 111111 | IGHV6-1 | IGHJ3 | CARGWGMVRSGWPTDLFDIW | IGKV3-20 | IGKJ1 | CQQYGTSPTF | Inf.Grp. |
| 111111 | IGHV6-1 | IGHJ3 | CARGWGLVRSGWPTDLFDIW | IGKV3-20 | IGKJ1 | CQQYGASPTF | Inf.Grp. |
| 111111 | IGHV6-1 | IGHJ3 | CARGWGMVRSGWPTDLFDMW | IGKV3-20 | IGKJ1 | CQQYGSSPTF | Inf.Grp. |
| 111111 | IGHV6-1 | IGHJ3 | CARGWGLVRSGWPTDLFDIW | IGKV3-20 | IGKJ1 | CQQYGTSPTF | Inf.Grp. |
| 111111 | IGHV6-1 | IGHJ3 | CARGWGMARSGWPTDLFDIW | IGKV3-20 | IGKJ1 | CQQYGTSPTF | Inf.Grp. |
| 111111 | IGHV6-1 | IGHJ3 | CARGWGMVRSGWPTDLFDMW | IGKV3-20 | IGKJ1 | CQQYGSSPTF | Inf.Grp. |
| 111111 | IGHV6-1 | IGHJ3 | CARGWGMVRSGWPTDLFDIW | IGKV3-20 | IGKJ1 | CQQYGNSPTF | Inf.Grp. |
| 111111 | IGHV6-1 | IGHJ3 | CARGWGMVRSGWPTDLFDVW | IGKV3-20 | IGKJ1 | CQQYGTSPTF | Inf.Grp. |
| 111111 | IGHV6-1 | IGHJ3 | CARGWGMVRSGWPTDLFDFW | IGKV3-20 | IGKJ1 | CQQYGTLPTF | Inf.Grp. |
| 111111 | IGHV6-1 | IGHJ3 | CVRAWGMVRSGWPTDLFDLW | IGKV3-20 | IGKJ1 | CQQYGTSPTF | Inf.Grp. |
| 111111 | IGHV6-1 | IGHJ3 | CVRSWGVVRSGWPTDLFDIW | IGKV3-20 | IGKJ1 | CQQYGTSPTF | Inf.Grp. |
| 111111 | IGHV6-1 | IGHJ3 | CARGWGMVRSGWPTDLFDIW | IGKV3-20 | IGKJ1 | CQQYGTLPTF | Inf.Grp. |
| 111111 | IGHV6-1 | IGHJ3 | CARAWGMVRSGWPTDLFDLW | IGKV3-20 | IGKJ1 | CQQYGTSPTF | Inf.Grp. |
| 111111 | IGHV6-1 | IGHJ3 | CARGWGMVRSGWPTDLFDIW | IGKV3-20 | IGKJ1 | CQQYGTSPTF | Inf.Grp. |
| 111111 | IGHV6-1 | IGHJ3 | CARGWGMVRSGWPTDLFDIW | IGKV3-20 | IGKJ1 | CQQYGTSPTF | Inf.Grp. |
| 111111 | IGHV6-1 | IGHJ3 | CARGWGMVRSGWPTDLFDIW | IGKV3-20 | IGKJ1 | CQQYGTSPTF | Inf.Grp. |
| 111111 | IGHV6-1 | IGHJ3 | CARGWGMVRSGWPTDLFDIW | IGKV3-20 | IGKJ1 | CQQYGTSPTF | Inf.Grp. |
| 111111 | IGHV6-1 | IGHJ3 | CARAWGMVRSGWPTDLFDVW | IGKV3-20 | IGKJ1 | CQQYGTSPTF | Inf.Grp. |
| 111111 | IGHV6-1 | IGHJ3 | CARGWGMVRSGWPTDLFDMW | IGKV3-20 | IGKJ1 | CQQYGSSRTF | Inf.Grp. |
| 111111 | IGHV6-1 | IGHJ3 | CARGWGMVRSGWPTDLFDIW | IGKV3-20 | IGKJ1 | CQQYGTSPTF | Inf.Grp. |
| 111111 | IGHV6-1 | IGHJ3 | CARGWGMVRSGWPTDLFDIW | IGKV3-20 | IGKJ1 | CQQYGASPTF | Inf.Grp. |

Table S2 The detailed information of clonotype cluster 149, 233,263

| **clonotype cluster id** | **IGHV** | **IGHJ** | **CDRH3** | **IGK/LV** | **IGK/LJ** | **CDRK/L3** | **sample** |
| --- | --- | --- | --- | --- | --- | --- | --- |
| 4 | IGHV4-59 | IGHJ4 | CARGFDFW | IGKV3-20 | IGKJ1 | CHQYGSSPWTF | Vac.Rec.Grp. |
| 4 | IGHV4-59 | IGHJ4 | CARGFEYW | IGKV3-20 | IGKJ1 | CQQYGSSPWTF | Unvac.Rec.Grp. |
| 4 | IGHV4-59 | IGHJ4 | CARGFDYW | IGKV3-20 | IGKJ1 | CQQYGSSPWTF | Inf.Grp.SCoV13 |
| 4 | IGHV4-59 | IGHJ4 | CARGFDYW | IGKV3-20 | IGKJ1 | CQQYGSSPWTF | Inf.Grp.SCoV13 |
| 4 | IGHV4-59 | IGHJ4 | CARGFDYW | IGKV3-20 | IGKJ1 | CQQYGSSPWTF | Inf.Grp.SCoV13 |
| 4 | IGHV4-59 | IGHJ4 | CARGFDYW | IGKV3-20 | IGKJ1 | CQQYGSSPWTF | Inf.Grp.SCoV13 |
| 4 | IGHV4-59 | IGHJ4 | CARGFDYW | IGKV3-20 | IGKJ1 | CQQYGSSPWTF | Inf.Grp.SCoV13 |
| 4 | IGHV4-59 | IGHJ4 | CARGFDYW | IGKV3-20 | IGKJ1 | CQQYGSSPWTF | Inf.Grp.SCoV13 |
| 4 | IGHV4-59 | IGHJ4 | CARGFDYW | IGKV3-20 | IGKJ1 | CQQYGSSPWTF | Inf.Grp.SCoV13 |
| 4 | IGHV4-59 | IGHJ4 | CARGFDYW | IGKV3-20 | IGKJ1 | CQQYGSSPWTF | Inf.Grp.SCoV13 |
| 4 | IGHV4-59 | IGHJ4 | CARGFDYW | IGKV3-20 | IGKJ1 | CQQYGSSPWTF | Inf.Grp.SCoV13 |
| 256 | IGHV3-73 | IGHJ4 | CTTALDYW | IGLV3-25 | IGLJ3 | CQSADSTNSYRVF | Inf.Grp.SCoV1 |
| 256 | IGHV3-73 | IGHJ4 | CTTALDYW | IGLV3-25 | IGLJ3 | CQSADRSNTYRVF | Inf.Grp.SCoV1 |
| 256 | IGHV3-73 | IGHJ4 | CTTALDYW | IGLV3-25 | IGLJ3 | CQSADNSGTYRVF | Inf.Grp.SCoV1 |
| 256 | IGHV3-73 | IGHJ4 | CTTALDYW | IGLV3-25 | IGLJ3 | CQSADNSNTYRVF | Inf.Grp.SCoV1 |
| 256 | IGHV3-73 | IGHJ4 | CTTALDYW | IGLV3-25 | IGLJ3 | CQSADDSSTYRVF | Inf.Grp.SCoV1 |
| 256 | IGHV3-73 | IGHJ4 | CTTALDYW | IGLV3-25 | IGLJ3 | CQSADNSGTYRVF | Inf.Grp.SCoV11 |
| 340 | IGHV3-23 | IGHJ4 | CAKDKTPDGVYDVDYW | IGKV2D-28 | IGKJ4 | CMQALQTPVTF | Hlty.Grp. |
| 340 | IGHV3-23 | IGHJ4 | CAKDKIPDGLYDLDYW | IGKV2D-28 | IGKJ4 | CMQALQTPLTF | Vac.Hlty.Grp. |
| 340 | IGHV3-23 | IGHJ4 | CAKDRLPDGLWDIDFW | IGKV2D-28 | IGKJ4 | CMQGLQTPLTF | Vac.Hlty.Grp. |
| 340 | IGHV3-23 | IGHJ4 | CAKDRTPDGLYDIDYW | IGKV2D-28 | IGKJ4 | CMQALQTPLTF | Vac.Hlty.Grp. |
| 340 | IGHV3-23 | IGHJ4 | CAKDLVPDGRWEIDIW | IGKV2D-28 | IGKJ4 | CMQGVQTPLTF | Vac.Rec.Grp. |
| 340 | IGHV3-23 | IGHJ4 | CAKDRTPDGLWDIDYW | IGKV2D-28 | IGKJ4 | CMQALQTPLTF | Vac.Hlty.Grp. |
| 340 | IGHV3-23 | IGHJ4 | CAKDRLPDGLWEIDLW | IGKV2D-28 | IGKJ4 | CMQGLQPPLTF | Vac.Hlty.Grp. |
| 758 | IGHV2-26 | IGHJ6 | CARIQREVTIFGVRYYYYGMDVW | IGLV1-51 | IGLJ3 | CGTWDSSLSAGVF | Inf.Grp.SCoV1 |
| 758 | IGHV2-26 | IGHJ6 | CARIQREVTIFGVRYYYYGMDVW | IGLV1-51 | IGLJ3 | CGTWDSSLSAGVF | Inf.Grp.SCoV11 |
